# Commentary on ‘the review on the progress in the surgical treatment of sacrococcygeal pilonidal sinus’

**DOI:** 10.1097/JS9.0000000000000768

**Published:** 2023-09-21

**Authors:** Hongru Sun, Dengfeng Yu

**Affiliations:** Department of General Surgery, Dalian University Affiliated Xinhua Hospital, Dalian, People’s Republic of China

*Dear Editor,*

We have read the review on the progress in the surgical treatment of sacrococcygeal pilonidal sinus (SPS)^1^. The subject of the manuscript is interesting and topical. The incidence of SPS is increasing year by year^2^. There are numerous treatment options for SPS, but no consensus has been reached on the preferred method of treatment. Postoperative recurrence and infection are indeed issues that warrant consideration.

The authors evaluated 54 studies and included 3612 patients in their meta-analysis. The review of the medical literature was rigorously conducted^1^. The authors concluded that the midline closure technique has a much higher incidence of postoperative recurrence and infection than other techniques. Moreover, the author emphasized the importance of adopting a comprehensive approach in formulating individualized solutions that best fit the patient’s needs^1^.

At the same time, the article also mentioned an interesting phenomenon that different researchers may have contradictory results using the same surgical method, and this phenomenon is particularly prominent in the study of minimally invasive surgery for treating SPS. As analyzed by the authors, studies of minimally invasive surgical treatment of SPS that contain complex SPS may increase postoperative recurrence and infection rates^1^. Therefore, preoperative evaluation of SPS is particularly important; MRI plays an important role in accurately measuring the basic parameters of SPS (size, volume, location, surrounding tissue), providing surgical access, and evaluating the risk factors for postoperative complications and recurrence^3^.

Surgeons typically utilize two-dimensional (2D) MRI to understand the nature of the lesion. However, through the use of 3D reconstruction technology, surgeons can obtain a clearer image of the relevant anatomical structures. In view of the high similarity between pilonidal sinus, anal fistula and perianal abscess, we refer to the method of Wiesław Guz^4^, MRI of the sacrococcygeal region of one SPS patient was reconstructed using 3D Slicer v. 5.2.2. By setting the parameters, 3D Slicer can automatically segment SPS, abscesses and surrounding infected tissues, and manually segment bones, muscles, skin, anal canal and rectum. And marked with different colors: green – SPS and subcutaneous abscess; red – muscle; blue – potentially infected tissue; white – bone; light red – anal and rectum; brown – skin. This is shown in Figure 1. After 3D reconstruction, it is easier to observe the relationship between infection and surrounding tissues by adjusting the transparency. We can clearly see: SPS formation at the posterior level of the coccyx; subcutaneous abscess formation at the sacrococcygeal region; infection of bilateral gluteal fissure and levator ani muscle are more likely.

**Figure 1:**
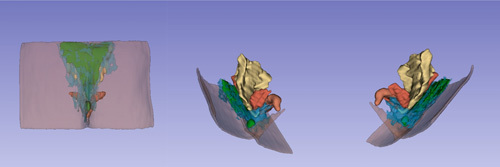
Selected layers from MRI examination for one of the patients. The individual structures of the sacrococcygeal region were marked with different colors (green: SPS and subcutaneous abscess; red: muscle; blue: potentially infected tissue; white: bone; light red: anal and rectum; brown: skin).

Through the 3D reconstruction of SPS, all three parties involved in the SPS diagnosis and treatment process can benefit. For doctors, 3D images are more beneficial for the preoperative assessment of patient conditions and the design of surgical plans. They provide a realistic roadmap for doctors to follow during the surgical process, enabling the eradication of the lesion and the evaluation of the surgical procedure after the operation. This, in turn, enhances surgical safety and reduces the rates of postoperative recurrence and infection. For patients, it is important to enhance their understanding of their condition and to promote informed consent. Moreover, for the benefit of interns, it allows them to receive high-quality training while reducing any discomforts of patients both physically and psychologically during clinical teaching.

Our present study is the first application of 3D printing technology in the treatment of SPS. This innovative technique offers an intuitive advantage to surgeons, patients and interns, but the effect of it needs objective assessment. We believe that by conducting 3D reconstruction of SPS to develop and implement more individualized surgical protocols for patients, coupled with comprehensive postoperative care^5^ and standardizing the diagnostic and treatment process for SPS, we can reduce the rate of postoperative recurrence and infection, and promote the development of perioperative Enhanced Recovery After Surgery in SPS.

## Ethical approval

The local Bioethical Commission (Dalian University Affiliated Xinhua Hospital) was consulted on the project protocol, and we were advised that this manner of testing is routinely performed for patients with SPS disease without needing to apply for written permission from the Bioethical Committee.

## Consent

Patients gave written permission for the use of electronic processing of obtained images and 3D reconstruction of their MRI examination results. Written informed consent was obtained from the patient for the publication of this case report and accompanying images. A copy of the written consent is available for review by the Editor-in-Chief of this journal on request.

## Sources of funding

This study was sponsored by the Summit Program of the Dalian Municipal Health Commission in China.

## Author contribution

H.-R.S. and D.-F.Y. conceived the original idea, met the criteria for authorship established by the International Committee of Medical Journal Editors, and verified the validity of the results reported. Both authors read and approved the final manuscript.

## Conflicts of interest disclosure

The authors declare no conflicts of interest.

## Research registration unique identifying number (UIN)

The paper is a Commentary/Letter to the Editor.

## Guarantor

Both authors read and approved the final manuscript.

## Data availability statement

The paper is a Commentary/Letter to the Editor.

## Provenance and peer review

Not commissioned, externally peer-reviewed.
